# Factors Associated With the Suspension of Domiciliary Dental Care for Older Adults During the First Wave of the Coronavirus Disease 2019 (COVID-19) Pandemic: A Comparative Study in the Japanese Local Government

**DOI:** 10.7759/cureus.70376

**Published:** 2024-09-28

**Authors:** Yuki Egashira, Yu Kubota, Ryo Watanabe

**Affiliations:** 1 School of Health Innovation, Kanagawa University of Human Services, Kanagawa, JPN

**Keywords:** adherence, claims data, covid-19, domiciliary dental care, older adults

## Abstract

Objective

The novel coronavirus has reduced the availability of dental services, including domiciliary dental care. The suspension of dental treatment worsens the dental condition. However, the impact on domiciliary dental care during the coronavirus disease 2019 (COVID-19) pandemic remains unclear. Therefore, the aim of this study is to determine the impacts of the COVID-19 pandemic on domiciliary dental care and associated factors.

Materials and methods

This comparative study used Japanese prefectural claims data, including data from older adults who utilized domiciliary dental care. We compared the suspension of dental treatment during the firstwave of the COVID-19 pandemic with that before the COVID-19 pandemic. The outcome was the completion of four consecutive months of treatment. The chi-squared test, t-test, and logistic regression were applied.

Result

Approximately 23.4% of participants suspended home dental services during the first wave of the COVID-19 pandemic, whereas 11.7% suspended these services before COVID-19. Logistic regression clarified the effect of the following variables (during the first wave of COVID-19 vs. before COVID-19): care need level of long-term care was 1-5 (=severe) (odds ratio (OR): 0.92 vs. no significance (NS)), visit by hospital dentists (OR: 1.83 vs. NS), volume of patients in the visited place (2-9 people, OR: NS vs. 0.72; over 10 people, OR: 0.58 vs. 0.32), planned physicians' visit (OR: 0.71 vs. 0.77), and taxed income per population (OR: NS vs. 1.00).

Discussion

Domiciliary dental care, especially those in care facilities, were a vulnerable population during the pandemic. This may contribute to the delivery of domiciliary dental care during any future pandemics.

## Introduction

The novel coronavirus, which emerged in Wuhan, China, in December 2019, spread globally, and almost 118,000 people were infected from March 11, 2020, in over 110 countries, and 4,291 patients died. Accordingly, the World Health Organization declared a "pandemic," indicating a global outbreak [1].

Many countries have implemented countermeasures to reduce the impact of these outbreaks, such as lockdowns that prohibit people from transferring. These countermeasures and the pandemic led to the government and academic organizations requesting the postponement or cancellation of non-urgent treatment. Consequently, a sizeable reduction in non-urgent operations and hospitalizations was observed in many countries [2,3]. In addition to medical services, home care services experienced reductions or cancellations [4].

Aside from medical services, the Japanese academician group reported that dental services of outpatients and domiciliary dental care were declined [5].

Reportedly, patients receiving domiciliary dental care are a vulnerable population because of the high care need for oral health and poor access to dental services [6-9]. The suspension of dental treatment worsens dental conditions, which causes serious comorbidities, increases the mortality rate, and decreases the quality of life (QOL) [10-12]. Therefore, continuous dental treatment is essential in frail populations. However, the quantitative impacts on domiciliary dental care during the coronavirus disease 2019 (COVID-19) pandemic have not been elucidated.

Among the Japanese 47 prefectures, Kanagawa is highly representative because it has the third-largest older population aged over 75 years. The first Japanese patient with COVID-19 was reported in the Kanagawa Prefecture [13]. Melby et al. clarified that municipalities in Norway with higher levels of contagion experienced adverse consequences for users of home medical care, compared with those with lower levels of contagion [14]. Therefore, the Kanagawa Prefecture may have a stronger burden on the dental healthcare system, compared with the other Japanese prefectures. We hypothesized that, during the COVID-19 pandemic, larger visited places would be less likely to accept domiciliary dental care because a survey of Japanese research projects showed that facilities for older adults canceled visits by dentists due to the risk of infection [5].

Thus, this study aimed to investigate these impacts and associated factors using claims data of older Japanese patients.

## Materials and methods

Japanese individuals aged 75 years and over are obligated to enroll in elderly insurance (EL); the enrolled individuals pay a 10% fee for services as a duty, which covers medical, dental, and pharmaceutical services. As a unique medical system, the Japanese public insurance covers dental treatment [15]. In long-term care (LTC), insurance is provided for home care services for older adults, which is also a legal duty. This comparative study used the Kokuho Database System (KDB) comprising the claims data of 75 Japanese older patients who participated in EL in the Kanagawa Prefecture. The coverage ratio was >99%, except for public assistance. The KDB is a Japanese database for the management of claims data, which comprises medical, dental, pharmaceutical, LTC claims, and health checkup data. The care need level of LTC consisted of five ranks (1=low and 5=high care needs) in accordance with the participant's physical status. These secondary data contained the provided medical services, with date, quantities, hospitals' codes, and patients' information including birthday, insured number, and care need level of LTC. All unique data were assigned anonymous identification numbers. Our study combined the dental, medical, and LTC data based on these identification numbers. Similar to the present study, the KDB was used to clarify large-scale epidemiological questions [16,17].

The study population was aged over 75 years; they were enrolled in EL and utilized domiciliary dental care (in patients' homes or facilities) during the first wave of the COVID-19 pandemic (April to May 2020). The study utilized 28 municipalities' data out of 33 in the Kanagawa Prefecture. The comparative study period was before the emergence of COVID-19 (July to October 2019). We defined our study population based on the fee utilized for domiciliary dental care (C000), which was claimed on the delivery of a planned dental visit. Furthermore, our patients were diagnosed with periodontal disease, defined as "K00-14, Diseases of the oral cavity, salivary glands and jaws [K code]" in the International Classification of Diseases, Tenth Edition [18].

**Figure 1:**
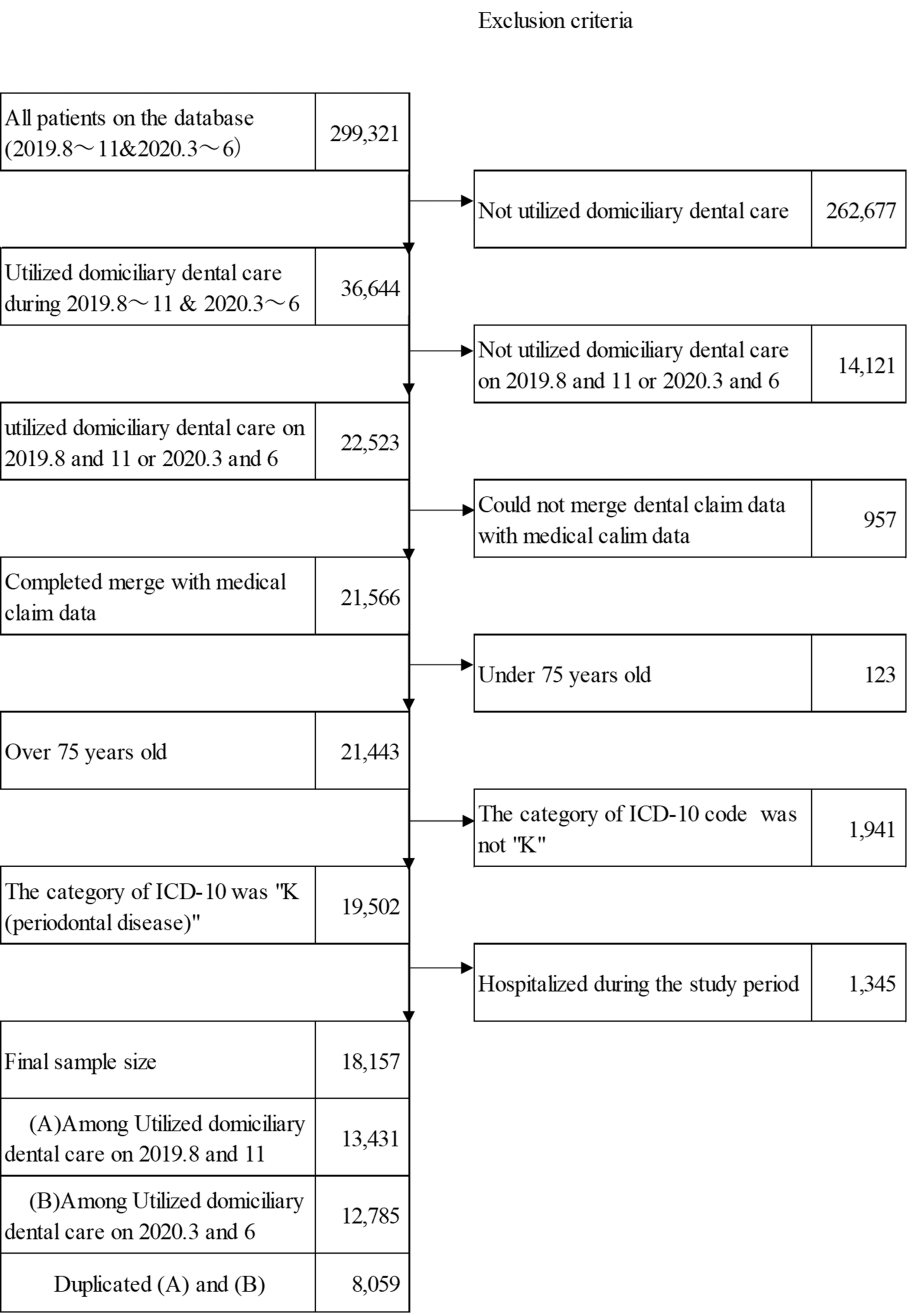
Study flowchart

We excluded the following individuals (Figure 1): those whose dental, medical, and LTC data could not be merged based on the identification number; those who were hospitalized during the study period; those who were diagnosed with COVID-19 during the study period; and those who had the first visit during the study period.

**Figure 2:**
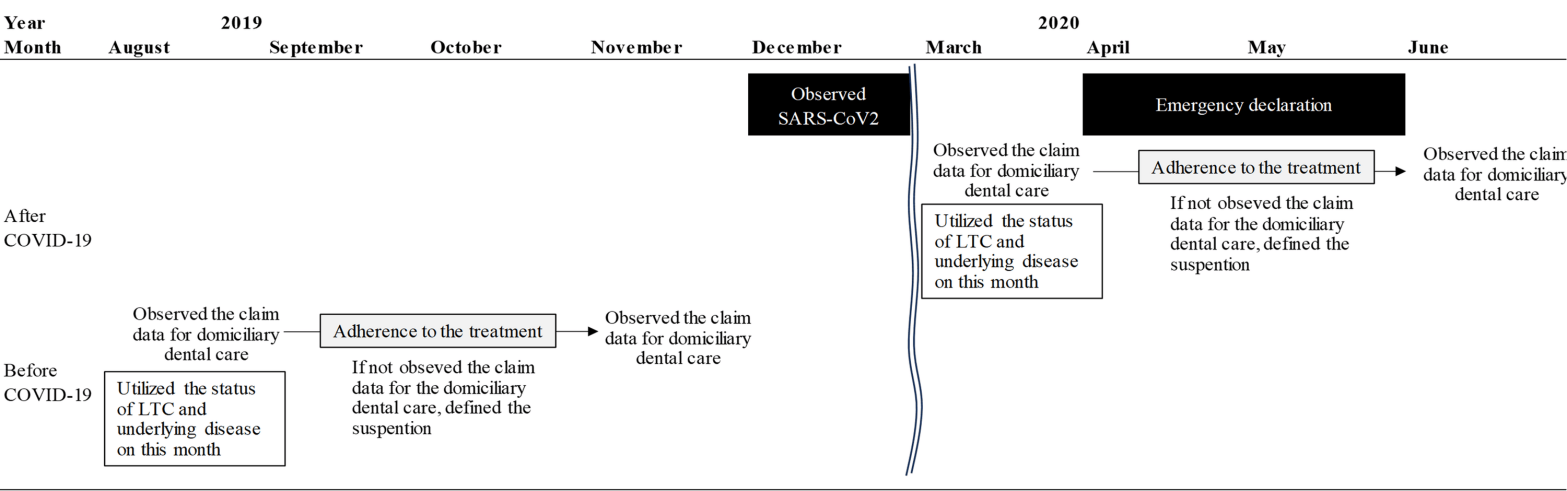
Study image

The outcome was domiciliary dental care for four consecutive months during the study period (Figure 2).

Our study focused on home dental or medical services and patient risks during the COVID-19 pandemic. Thus, the explanatory variables were utilizing dental or medical services, which were "domiciliary dental care (C000)," including the volume of patients in each place (1, 2-9, or over 10) and service providers (clinics or hospitals). Moreover, we included the status of LTC provided for five-level physical status and comorbidities: diabetes, hypertension, dementia, renal failure, and cancer. Additionally, in cases where patients received home care via physician visits (C002, C002-2) or nursing station care (C007), we inferred that patients highly accepted domiciliary dental care because of medical and dental corroboration, such as the exchange of information. A Japanese nested case-control study of homebound older adults reported that physician visits were positively associated with domiciliary dental care [19]. Other Japanese researchers have insisted on the importance of collaboration between domiciliary dental care and nursing stations [20]. Further, we considered high-risk populations for COVID-19, such as those with high care needs, those with comorbidities, and those who refused domiciliary dental care to avoid infection [21,22].

Based on previous studies, we set the following covariates to adjust for confounders: patient age, sex, number of dentists in each region per population, and taxed income per population [20,23-25].

We set the outcome as completing four consecutive months of treatment ("1" indicated completion of treatment). The chi-squared test and t-test were applied as appropriate. After calculating basic statistics, logistic regression was conducted. The statistically significant level was set at 5%. The statistical software used was Stata MP 16.1 (StataCorp LLC, College Station, Texas, United States).

This study was approved by the Ethics Committee of Kanagawa University of Human Services (SHI No. 68). Patient data were not extracted as anonymized data, so informed consent was not required.

## Results

Flowchart of the study population

Figure 1 illustrates the flowchart of the study population. We identified 12,785 individuals enrolled in EL and utilizing domiciliary dental care during the COVID-19 pandemic and 13,431 who utilized such services before the COVID-19 pandemic.

The result of the suspension

Table 1 indicates that 23% (2,941) of the participants suspended their home dental services during the first wave of the COVID-19 pandemic, whereas the corresponding percentage for the comparative period was 11.7% (1,569).

**Table 1:** The result of the suspension to domiciliary dental care during the emergency declaration p<0.01 COVID-19: coronavirus disease 2019

Period/suspended or not	Not suspended	Suspended
After COVID-19 (2020.3-6)	77%	23%
Before COVID-19 (2019.8-11)	88.3%	11.7%

Basic characteristics

Table 2 lists basic statistics. Among the analyzed variables, average age, comorbidities, and the utilization of home medical services and nursing station services significantly differed between the two periods.

**Table 2:** Basic statistics COVID-19: coronavirus disease 2019; SD: standard deviation; *1: t-test; *2: chi-squared test; *3: the number at the regional level

Data/period		The duration of domiciliary dental care		
		After COVID-19 2020.3-6	Before COVID-19 2019.8-11	P-value
		N=12,785	N=13,431	
Patients' status				
Average age^*1^		86.89	87.20	<0.01
SD		5.84	5.94	
Gender^*2^				
Male		24%	23.4%	0.233
Female		76%	76.6%	
Level of long-term care^*2^				
Not certified		6.7%	6.3%	0.434
Low		3.4%	3.5%	
Severe		89.9%	90.2%	
Comorbidities^*2^				
Yes		85.3%	84.6%	0.116
No		14.7%	15.4%	
Domiciliary dental care				
Provider type^*2^				
Hospital		0.2%	0.3%	0.064
Clinic		99.8%	99.7%	
Patients' volume of visited place^*2^				
1		15.5%	15.1%	0.641
2-9		32.3%	32.5%	
Over 10		52.2%	52.4%	
Medical services				
Planed physicians' visit^*2^				
Provided		50.5%	47.9%	<0.01
Not provided		49.5%	52.1%	
Nursing stations' services^*2^				
Provided		6.6%	6.2%	0.112
Not provided		85.3%	84.6%	
Social economic status and medical resources^*3^				
Taxed income per population^*1^		3,630.97	3,629.57	0.764
SD		377.42	378.22	
Average family members^*1^		2.13	2.13	<0.01
SD		0.93	0.93	
Number of dentists per population^*1^		73.01	73.14	0.505
SD		15.18	15.32	

The association with suspension of domiciliary dental care

Table 3 presents the results of logistic regression analyses. Our study identified the following significant variables (during the first wave of COVID-19 vs. before the COVID-19 pandemic): care need level of LTC (=severe) (odds ratio (OR): 0.77 vs. no significance (NS)), visit by hospital dentists (OR: 2.65 vs. 2.97), volume of patients in the visited place (2-9 people, OR: NS vs. 0.76; over 10 people, OR: 0.58 vs. 0.33), planned physicians' visit (OR: 0.70 vs. 0.75), and taxed income per population (OR: NS vs. 1.00).

**Table 3:** The result of logistic regression COVID-19: coronavirus disease 2019; CI: confidence interval; *1: diabetes, hypertension, dementia, renal failure, and cancer; *2: the number at the regional level

Data/period			After COVID-19 (N＝12,785)				Before COVID-19 (N＝13,431)			
			Odds ratio	P-value	95% CI		Odds ratio	P-value	95% CI	
Patients' status										
Age			1.00	0.22	1.00	1.01	1.00	0.90	0.99	1.01
Gender (reference=male)										
Female			0.98	0.64	0.89	1.08	1.02	0.76	0.90	1.16
Level of long-term care (reference=not certified)										
Low			0.98	0.89	0.75	1.28	0.93	0.70	0.65	1.33
Severe			0.77	0.00	0.65	0.90	0.96	0.72	0.76	1.20
Comorbidities^*1^			0.95	0.43	0.85	1.07	1.11	0.20	0.95	1.29
Domiciliary dental care										
Provider type (reference=clinic)										
Hospital			2.65	<0.01	1.27	5.54	2.97	<0.01	1.59	5.56
Patients' volume of visited place (reference=1)										
2-9			0.94	0.38	0.83	1.07	0.76	<0.01	0.65	0.88
Over 10			0.58	<0.01	0.51	0.65	0.33	<0.01	0.29	0.39
Medical services										
Planed physicians' visit (reference=not provided)			0.70	<0.01	0.64	0.76	0.75	<0.01	0.67	0.84
Nursing stations' services (reference=not provided)			0.95	0.61	0.80	1.14	1.05	0.68	0.85	1.29
Social economic status and medical resources^*2^										
Taxed income per population			1.00	0.57	1.00	1.00	1.00	0.01	1.00	1.00
Average family member			0.94	0.82	0.56	1.58	1.63	0.16	0.83	3.22
Number of dentists per population			1.00	0.49	1.00	1.00	1.00	0.70	1.00	1.01
Cons			0.63	0.54	0.14	2.77	0.03	<0.01	0.00	0.21

## Discussion

We investigated the impact of the first wave of the COVID-19 pandemic on domiciliary dental care, utilizing claims data in the Kanagawa Prefecture, which is an area in Japan that was majorly affected during the pandemic. Overall, our study showed that the initial phase of the COVID-19 pandemic reduced adherence to domiciliary dental care. Moreover, the number of patients in the visited place had the most notable impact on domiciliary dental care.

The most interesting finding is that the volume of patients in the visited place (over 10 people) was associated with a 75.6% higher OR for the suspension of dental services during the first wave of the COVID-19 pandemic, compared to before the COVID-19 pandemic. A potential explanation cited in a Japanese research article [5] is that these facilities suspended home dental care to mitigate the risk of infection. This result is partly consistent with those of previous studies. The Canadian study by Emily et al., which surveyed home care clients and providers, noted a decrease in home care services due to cancellations by clients (99.3%) [26]. This implies that the impact of cancellations by healthcare providers is minor. We can assume that Japan may have faced a similar situation because of the policy of the Japanese government. On April 1, 2020, a Japanese expert committee advocated that three close situations, called "three Cs," namely, places with poor air condition, crowded places, and speaking to someone at a close distance simultaneously, cause a high risk of infection. Hence, this announcement to prevent cluster infections possibly led users or facilities to avoid utilizing home care services [27]. Another possible scenario is that patients who stayed in LTC facilities prioritized medical care over dental care. Soh showed that, in Singapore, older individuals who stayed at LTC facilities prioritized medical over dental care. The provision of medical treatment was 93%, whereas that of dental care was 70.7%. Actual offers of in-house medical services were 42.6%, whereas those of in-house dental services were 5.9% [28]. To summarize, the first wave of the COVID-19 pandemic may have impacted home dental care for patients residing in facilities. The potential reasons for this were considered as patient avoidance owing to the risk of infection and low prioritization of dental care.

The second finding was that physician visits contributed to continuous domiciliary dental care not only before the COVID-19 pandemic but also after it. One study showed that physician visits were positively associated with adherence to domiciliary dental care. A nested case-control study using nationwide LTC claims data conducted by Ishimaru et al. clarified that the OR of physician visits was 3.15, which was the third highest OR in their multivariable logistic regression analysis results [19]. The present study implies that cooperation between physicians and home care dentists is crucial, even during the pandemic. A possible explanation for this is that home care physicians understand the importance of dental care and advocate for their patients to receive domiciliary dental care. However, to the best of our knowledge, there is no evidence of an association between home medical services and dental services during the COVID-19 pandemic. Therefore, future studies should obtain concrete findings regarding this relationship.

The third finding was that a care need level of 1-5 (=severe) was negatively associated with the suspension of domiciliary dental care during the COVID-19 pandemic. In Japan, participants with high care needs can utilize LTC services free of cost, in accordance with their care need level. One Japanese study reported a low out-of-pocket rate because participants with high care needs in LTC were accustomed to domiciliary dental care [19]. However, this phenomenon was observed before the COVID-19 pandemic; therefore, the key implication is that patients with high care needs may have continued to receive domiciliary dental care during the COVID-19 pandemic. Moreover, some studies have identified this population as vulnerable. A Japanese survey of homebound older people indicated that individuals with low activities of daily living account for a high percentage of those in need of dental care, such as partial or full assistance with toothbrushing (37.6%), cleaning dentures (55.6%), and eating (46.7%) [7]. Noguchi et al. also documented that Japanese patients who visited nursing stations had low scores on the Japanese Oral Health Assessment Tool, an assessment tool for oral health in older adults. In particular, a care need level 3 or higher indicates a high risk for poor oral health [6].

Finally, domiciliary dental care provided by hospital dentists was more likely to be discontinued. This finding is consistent with those of other studies. A study in Taiwan conducted by Lee et al. showed that hospitals facing a decrease in dental visits accounted for 13.8%, whereas dental clinics accounted for only 4.3% [29]. However, in the present study, the percentage of participants utilizing domiciliary dental care by hospital dentists before the COVID-19 pandemic was only 1%. Therefore, we must focus on generalizing these results to other countries.

Importantly, even before the COVID-19 outbreak, the services provided to these populations were possibly insufficient. A French cross-sectional study of older adults revealed that 23% of respondents had unaddressed healthcare needs and the most prevalent need was dental treatment, which accounted for 17.7% of the unaddressed needs [30]. However, oral health needs to be given more attention because of the various disadvantages for patients. Bailey et al. insisted that issues with chewing and swallowing and mouth pain are risk factors for poor nutrition in older adults. Individuals with these issues tend to develop diseases due to the deficiencies of vitamins B-6 and A. In addition, participants with poor nutritional status were diagnosed with twice the number of diseases compared with those without oral health concerns [10]. An epidemiological study conducted in Stockholm between 1971 and 1996 indicated that poor oral health was positively associated with mortality rate, despite eliminating patients who died of cardiovascular disease from the study [11]. Furthermore, Kandelman et al. discussed that poor oral health is strongly related to diabetes and directly influences older people's QOL [12]. Avlund et al., in contrast, argued that oral impairments such as having no teeth, oral functional limitations such as chewing problems, and general functional limitations such as mobility problems were interrelated [31]. Therefore, we should explore the risk of infection and worsened dental conditions due to the suspension of home dental care.

This study has a few limitations. Firstly, we could not consider some sociodemographic factors, such as patient education or family income level, owing to the limitations of our dataset. A Japanese study reported a positive association between neighborhood income and receipt of domiciliary dental care. Thus, we may have underestimated or overestimated the impact of the variables in our logistic regression model. Secondly, we could not determine the types of visited places because the claims data could only determine the number of patients at each place. Thus, the definition of a facility in our study was based on the number of patients. We possibly misunderstood that places with over 10 patients were full facilities, while some of them were giant apartment houses, and over 10 patients lived in these houses. Lastly, we could not clarify causality because this was a retrospective observational study. Our findings are only regarding associations with treatment suspension.

## Conclusions

This is the first study to clarify the factors associated with the suspension of domiciliary dental care during the initial phase of the COVID-19 pandemic, utilizing large-scale dental claims data. In conclusion, the suspension of domiciliary dental care increased during the COVID-19 pandemic, and the volume of patients at the visited place was the most notable factor associated with the suspension of domiciliary dental care, compared with other variables. These findings may contribute to improving the delivery of domiciliary dental care during future pandemics.
